# Development and Evaluation of a Bicistronic DNA Vaccine against Nervous Necrosis Virus in Pearl Gentian Grouper (*Epinephelus lanceolatus* × *Epinephelus fuscoguttatus*)

**DOI:** 10.3390/vaccines10060946

**Published:** 2022-06-14

**Authors:** Tianwen Lin, Jing Xing, Xiaoqian Tang, Xiuzhen Sheng, Heng Chi, Wenbin Zhan

**Affiliations:** 1 Laboratory of Pathology and Immunology of Aquatic Animals, KLMME, Ocean University of China, No. 5 Yushan Road, Qingdao 266003, China; tianwenlin23@163.com (T.L.); tangxq@ouc.edu.cn (X.T.); xzsheng@ouc.edu.cn (X.S.); chiheng@ouc.edu.cn (H.C.); wbzhan@ouc.edu.cn (W.Z.); 2 Laboratory for Marine Fisheries Science and Food Production Processes, Qingdao National Laboratory for Marine Science and Technology, No. 1 Wenhai Road, Aoshanwei Town, Qingdao 266071, China

**Keywords:** nervous necrosis virus, IRF3, pearl gentian grouper, bicistronic DNA vaccine, immune response, challenge

## Abstract

Nervous necrosis virus (NNV) can cause enormous economic losses in mariculture. Vaccines are promising ways to control the disease. In this study: the interferon regulatory factor 3 (IRF3) gene of pearl gentian grouper was cloned and functionally analyzed; then a bicistronic DNA vaccine encoding both capsid protein (CP) and IRF3 was constructed; then the cellular, humoral, and local immune responses in the grouper after immunization were investigated; and then the protective effects after the NNV challenge were investigated. The results showed that the vaccine successfully expressed CP and IRF3. After immunization, the lymphocytes were recruited at the injection site in the muscles. The percentage of sIgM+ lymphocytes in the head, kidney, and spleen significantly increased and peaked at 28.8 ± 3.1% and 42.6 ± 4.2% at the 3rd to 4th weeks. Six immune-related genes were significantly up-regulated. In the meantime, the total antibodies, anti-NNV specific antibodies, and neutralizing antibody titers in serum increased. After the challenge with 10^5^, 10^6^ or 10^7^ TCID_50_/fish, the relative percent survival rate was 81.25%, 73.91%, and 66.67%, respectively. In 10^6^ TCID_50_/fish groups, the percentages of sIgM+ lymphocytes, antibodies, and the viral load were investigated. In conclusion, the bicistronic vaccine significantly induced humoral and cellular responses in pearl gentian grouper and provided effective protection against NVV infection.

## 1. Introduction

The grouper is a marine economic fish species in China. In 2020, the production of grouper was 192,045 tons, ranking third in China’s marine fish aquaculture production [1]. In recent years, the fish culture industry is severely affected by viral nervous necrosis (VNN), also known as viral encephalopathy and retinopathy (VER), which has become one of the most serious epidemic diseases worldwide. Nervous necrosis virus (NNV), and the pathogen of this disease belongs to, *Nodaviridae Betanodavirus*, could infect more than 177 species of cultured marine and freshwater fish [2]. The virus mainly infects the nervous system of aquatic animals, especially in the larval and juvenile stages of fishes (about 0.1–15g). Therefore, high mortalities caused by this virus have also been reported [3].

The virus is a nonenveloped virus consisting of two genome segments: RNA1 and RNA2. RNA1 encodes the viral part of the RNA-dependent RNA polymerase (RdRp), whereas RNA2 encodes the coat protein (CP), which is also the main immunogenic protein of the virus [4,5]. There are four reported genotypes: red-spotted grouper nervous necrosis virus (RGNNV), barfin flounder nervous necrosis virus (BFNNV), tiger puffer nervous necrosis virus (TPNNV), and striped jack nervous necrosis virus (SJNNV) [3,6,7]. NNV CNPgg2018, a genotype of RGNNV, was previously isolated from diseased pearl gentian grouper (*Epinephelus lanceolatus* × *Epinephelus fuscoguttatus*) and stored in our laboratory [8].

DNA vaccines against NNV have been reported in some species. In orange-spotted grouper (*Epinephelus coioides*), a DNA vaccine (based on CP) mixed with CpG oligodeoxynucleotide could induce the production of anti-NNV specific antibodies and the up-regulated expression of TLR9, Mx, and IL-1β, and then elicit a relative percent survival rate (RPS) of 47% [9]. An oral chitosan DNA vaccine-induced up-regulation of cell-mediated cytotoxicity and interferon genes in European sea bass (*Dicentrarchus labrax*) and elicited a RPS of 45% [10]. Chitosan-tripolyphosphate (CS/TPP) encapsulated oral DNA vaccine elicits a RPS of 60% in Asian sea bass (*Lates calcarifer*) [11]. In turbot (*Scophthalmus maximus*), a DNA vaccine based on CP elicited an inflammatory response in the muscle at the injection site, and a challenge at the 8th day post-immunization elicited a RPS of 18% [12]. Recently, the vaccines with both better immune and protective effects are still under investigation.

Interferon regulatory factor 3 (IRF3) plays a key role in innate responses against viruses [13]. There have been reports that IRF3 exerts antiviral function against NNV infection. In sea perch (*Lateolabrax japonicus*), the overexpression of IRF3 in vitro significantly inhibits virus replication and significantly upregulates IFN-I and IFN stimulated genes (ISGs) at the same time, which leads to the activation of apoptosis-related enzymes in the early stages of NNV infection [14]. In Asian sea bass, the expression of IRF3 is up-regulated, and IRF3 activates IFN-stimulated response elements (ISRE)/NF-κB promoters and regulates interferon, ISGs, and pro-inflammatory cytokine gene expression after NNV infection [15], suggesting it has potential as an adjuvant for viral vaccines.

In this study, the antiviral replication function of ELIRF3 (IRF3 of *Epinephelus lanceolatus*, the male parent of the pearl gentian grouper) was validated. Then a bicistronic DNA vaccine was constructed. After immunization, humoral immunity, cellular immunity, and local immunity in the muscles at the injection site elicited by the vaccine were investigated. Then after the challenge, the immune protection (including the percentage of sIgM+ lymphocytes, antibodies, and the viral load in target organs) invoked by the vaccine to the grouper was evaluated. Overall, the results showed solid data for a promising vaccine candidate. In the meantime, this study provided new insights for the development of NNV control.

## 2. Materials and Methods

### 2.1. Virus

Nodavirus (NNV, strain CNPgg2018, genotype RGNNV), previously isolated from diseased pearl gentian grouper, was propagated in the SSN-1 cell line. The cell line was cultured in Leibovitz’s L-15 medium (Gibco, Waltham, MA, USA) containing 10% fetal bovine serum (FBS) and 1% Penicillin-Streptomycin (Gibco, Waltham, MA, USA) at 28 °C. Virus was propagated and titrated in SSN-1 cells as described previously [8].

### 2.2. Antibodies and Animals

The antibody against immunoglobulin (IgM) of pearl gentian grouper was produced according to previous report [16]. Briefly, the serum of the grouper was purified by HiTrap™ Protein L resin prepacked column (GE Healthcare, Chicago, IL, USA) to obtain the purified IgM. Then the purified IgM was immunized to Balb/c mice. After immunization, the obtained antiserum of mice was purified with protein G-agarose column (Pierce/Thermo Scientific, Waltham, MA, USA) following the manufacturer’s instructions. The obtained purified antibody is the mouse anti-grouper IgM polyclonal antibody, named GIgM-Pab [17]. Mouse anti-NNV monoclonal antibody was stored in our laboratory [18].

Healthy juvenile pearl gentian grouper (3 ± 1 g) was purchased from a farm in Rizhao, Shandong Province, China. The fish were used for subsequent immunization and challenge experiments and reared in 15 L water tanks filled with fresh seawater at 28 ± 1 °C, and half of the seawater was replaced every day. All fish were fed commercial pellet diet at 5% body weight per day.

### 2.3. Cloning of ELIRF3 and Verification Its Antiviral Function

ELIRF3 was cloned by polymerase chain reaction (PCR) amplification with reference to the IRF3 gene sequence of *Epinephelus lanceolatus* (GenBank No. XM_ 033611218.1) using specific primers (Table 1). Then the protein domain of ELIRF3 was predicted by ExPASy software (smart.embl-heidelberg.de). Three dimensional models of proteins were elucidated by SWISS-MODEL [19]. Alignment of amino acid sequences and construction of phylogenetic tree (neighbor-joining—NJ method) were performed by MEGA5 software. Graphics are embellished via the iTOL server [20]. The Gene bank accession numbers of IRF3 of different species are as follows: *Epinephelus coioides* (AGC31487.1), *Oplegnathus fasciatus* (AHX37215.1), *Siniperca chuatsi* (XP_044035778.1), *Miichthys miiuy* (AHB59737.1), *Larimichthys crocea* (NP_001290316.1), *Danio rerio* (NP_001137376.1), Carassius auratus (ADO52204.1), *Ctenopharyngodon Idella* (AHL29306.1), *Gallus gallus* (NP_990703.2), *Xenopus laevis* (QYW22359.1), *Bos taurus* (NP_001025016.1), *Ovis aries* (XP_004015427.2), *Sus scrofa* (NP_998935.1), *Homo sapiens* (AAH71721.1), *Mus musculus* (NP_058545.1), and *Rattus norvegicus* (NP_001006970.1).

To verify the antiviral replication function of ELIRF3, the IRF3-ligated bicistronic plasmid and empty bicistronic plasmids were transfected with 70–80% SSN-1 cell line for 48 h, and then the cells were infected with 1 × 10^4^ TCID_50_ NNV and collected to extract RNA at 24/48 h, and the virus copy number was detected by quantitative real-time polymerase chain reaction (qPCR). In addition, the appearance of cytopathic effect (CPE) in cells was observed at 24 h after infection.

### 2.4. Bicistronic Plasmid Construction and Its Expression In Vivo and In Vitro 

The bicistronic plasmids were constructed as previously reported [21]. In short, the open reading frames of CP gene (GenBank No. MT157514.1) and IRF3 gene were amplified by PCR using respective specific primers (Table 1). Then, the PCR products of IRF3 were digested with NotI and kpnI restriction enzymes, and CP was digested with Hindll and EcoRI. The digested PCR products were inserted into the downstream of the cytomegalovirus immediate early (CMV) promoter and the downstream of the elongation factor 1α-subunit (EF-1α) promoter of the pBudCE4.1 vector to construct the bicistronic plasmids. PCR amplification and DNA sequencing (Tsingke, Qingdao, China) were used to determine the accuracy of the transgenes in the recombinant plasmids, the CP^+^-pBudCE4.1-IRF3^−^ (named C-P), CP^−^-pBudCE4.1-IRF3^+^ (named P-I), CP^+^-pBudCE4.1-IRF3^+^ (named C-P-I), and CP^−^-pBudCE4.1-IRF3^−^ (named P) were used as a negative control. Recombinant plasmids were extracted and endotoxins were removed with the EndoFree Plasmid Kit (Tiangen, Beijing, China). The successful expression of CP and IRF3 was verified by transfecting the recombinant plasmids into the hirame natural embryo cells (HINAE) cell line and the frozen section of the muscle of the fish injection site by immunofluorescence analysis (IFA) using the mouse anti-NNV monoclonal antibody/rabbit anti-His polyclonal antibody (Yeasen, Shanghai, China) and goat anti-mouse IgG-488/goat anti-rabbit IgG 649 as previously described [21,22].

### 2.5. Vaccination and Sampling

The concentration of plasmids was measured using Nanodrop 8000 spectrophotometer and adjusted to 200 ng/μL. Six hundred groupers were randomly divided into four groups (150 fish/group), and each fish was injected intramuscularly with 4 μg (20 μL, 1 μg/g of body weight) recombinant plasmids (C-P-I, C-P, P-I, and P) [9].

The spleen, head kidney, and muscle of injection sites were collected from three random fish in each group and placed in RNA later reagent (TaKaRa, Tokyo, Japan) on the 1st/3rd/5th day after immunization to analyze the relative expression of immune-related genes by qPCR. Moreover, the muscles on the 1st/3rd/5th/7th day after immunization were sampled for subsequent histopathological analysis.

Lymphocytes in the spleen (SPLs) and head kidney (HKLs), and serum of three random fish were isolated for 1–6 weeks after immunization. The lymphocytes were isolated as previously reported and used for subsequent flow cytometry (FCM) analysis [16]. For the isolation of serum, the fish (N = 3) were anesthetized with MS-222, the tail was severed, and the blood was collected through a capillary from the 1st to 6th weeks post vaccination. The blood was then centrifuged to obtain serum and aliquoted and stored at −80 °C until enzyme linked immunosorbent assays (ELISA) and neutralizing antibody titer tests were ready to be conducted.

### 2.6. Challenge and Sampling

The lethal dose of 50% (LD_50_) was 1.0 × 10^5^ TCID_50_, calculated according to previous report [23]. For NNV challenge studies, 90 fish from each group were randomly selected and divided into 3 subgroups, cultured in three tanks, and virus was administered intraperitoneally with a dose of cultured in three tanks, and virus administered intraperitoneally with doses of 1.0 × 10^5^, 10^6^, 10^7^ TCID_50_ live NNV per fish at the 6th week post-immunization [9]. Survival of each group was monitored over a period of 21 days after the challenge, and RPS was calculated according to the method of Amend et al. [24]. Meanwhile, the remaining individuals were all injected with 1.0 × 10^6^ TCID_50_/fish NNV for subsequent analysis of the immune protection, brains and eyes of three fishes were sampled and placed in RNA later reagent for qPCR analysis on the seventh day after the challenge, the serum, SPLs and HKLs from three random fish were collected as described above at 1–2 weeks after challenge for subsequent analysis.

### 2.7. Quantitative Real-Time Polymerase Chain Reaction

The qPCR procedure was used to analyze the expression of immune-related genes at spleen, head kidney, and the muscle of injection site, as well as the virus copy number in SSN-1 cells and the viral load in brains and eyes after NNV infection. According to the manufacturer’s instructions, total RNA was extracted using TRIZOL reagent (Baosheng, Dalian, China) from the tissues as previously reported [21].

An absolute fluorescence quantitative PCR standard curve for RNA2 has been established to quantify NNV copies [18]. Total RNA was quantified with Nanodrop 8000 spectrophotometer (Thermo Fisher Scientific, Waltham, MA, USA). The reverse transcription of RNA and the removal of genomic DNA (gDNA)were performed according to the manufacturer’s instructions using HiScript III RT SuperMix for qPCR (Vazyme, Nanjing, China). The expression profiles of immune-related genes (CD4, CD8α, IgM, MHC Iα, Mx, and TNF-α; primers were listed in Table 1) and virus copies (CP) were analyzed in a Light Cycler^®^ 480 II Real Time System (Roche, Basel, Switzerland) using SYBR Green I Master (Roche, Basel, Switzerland) by qPCR. The reaction program was set up according to the manufacturer’s instructions. The β-actin gene was used as an internal control, and each measurement was performed in triplicate. The expression of each gene relative to β-actin in each immune group was analyzed using the 2^−ΔΔCt^ method. The mRNA level in each tissue of the fish injected with pBudCE4.1 group was set to one.

### 2.8. Histological Examination

The muscle tissues of the immunized grouper on the 5th day after vaccination were sampled and histologically analyzed using hematoxylin and eosin (H&E) staining as previously reported [19]. In short, fresh samples were fixed with Bouin’s solution for 12–18 h, washed off with 70% alcohol, and then embedded in paraffin wax. The tissue was then sliced into 7 μm sections, transferred to pretreated microscope slides, and dried overnight at 37 °C. After dewaxing with xylene and a 50% xylene/ethanol solution, the sections were rehydrated by successive immersion in 95%, 80%, 70%, 50%, and 30% ethanol for five minutes. The sections were stained with hematoxylin for 10 min, differentiated with 0.1% acid alcohol for 45 s, and washed with water for 30 min. Subsequently, the sections were dehydrated by a series of ethanol solutions, counterstained with eosin for 45 s to reveal the cytoplasmic structure, and differentiated in 95% ethanol for 45 s. Finally, the slides were dehydrated twice with 100% ethanol, clarified in xylene, fixed with neutral balsam, and examined for histological changes using the Zeiss microscope (Oberkochen, Germany).

### 2.9. Flow Cytometry

The percentages of sIgM+ B lymphocytes in spleen and head kidney after immunization and challenge were investigated as described previously [16]. In short, to detect the percentage of sIgM+ B lymphocytes, the harvested lymphocytes were incubated with 500 μL GIgM-Pab (1:1000) for one hour at 37 °C. The cells were washed three times with phosphate buffered saline (PBS) and recovered by centrifugation at 680× *g* for 5 min. Then, Alexa Flour 488 conjugated goat anti-mouse IgG (1:1000, Thermo Fisher Scientific, Waltham, MA, USA) was added, and the samples were incubated at 37 °C in a dark place for one hour. The percentage of sIgM+ lymphocytes was analyzed by Accuri C6 cytometer. Mouse negative serum was used as a negative control.

### 2.10. Enzyme Linked Immunosorbent Assays

The level of specific antibodies against NNV and total antibodies in the serum were detected by ELISA. The results of the preliminary experiments show that the best dilution of serum is 1:60.

For the analysis of specific antibodies, 100 μL (10^3^ TCID_50_/mL) NNV, Bovine serum albumin (BSA) (4%), immunized fish serum (1:60), GIgM-pab (1:2000), and AP-conjugated goat-anti-mouse IgG (1:3000) were sequentially coated in 96-well plates. For the analysis of total antibodies, 100 μL serum (1:60), BSA (4%), GIgM-pab (1:2000), and AP-conjugated goat-anti-mouse IgG (1:3000) were sequentially coated in 96-well plates. All coating processes were carried out at 37 °C. After each step of coating, the well plate was washed three times with PBS containing 0.05% (*v*/*v*) Tween-20 (PBST). Finally, p-nitrophenyl phosphate (pNPP, Sigma, St. Louis, Missouri, USA) was added to initiate a color reaction, and the plates were incubated for 30 min in the dark at room temperature. The absorbance at 405 nm was then measured by ELISA reader (TECAN, Männedorf, Switzerland). Serum from fish injected with pBudCE4.1 was a negative control.

### 2.11. NNV Neutralization Assay

Serum from immunized and non-immunized fish was used to analyze their neutralizing activity against NNV. The grouper serum was sterilized with a 0.22-μm filter and serially diluted two-fold (from 1:8 to 1:1024) in L-15 medium. SSN-1 cells were cultured as a monolayer (80% confluent) on a 96-well plate. Fifty μL of serially diluted serum was mixed with the same volume of NNV (10^4^ TCID_50_/mL) at 28 °C for two hours. Then SSN-1 cells were incubated with the mixture for 1.5 h. The infected SSN-1 cells were washed three times with sterile PBS and then cultured with L-15 medium containing 2% FBS for six days. Each serum dilution was repeated in three parallel wells. The number of wells exhibiting the CPE was monitored, and the titer of neutralizing antibody was determined by obtaining the highest dilution that reduces CPE by approximately 50% compared with the virus control group.

### 2.12. Statistical Analysis

GraphPad Prism 9 was used to perform statistical analysis (GraphPad software, Inc. San Diego, CA, USA). All data were displayed as mean ± standard deviation (SD). Differences treated groups in FCM, ELISA, and qPCR were analyzed by analysis of variance (ANOVA). Compared with the pBudCE4.1 group, the statistical difference between the fish inoculated with P-I, C-P, and C-P-I was carried out to the level of * *p* < 0.05, ** *p* < 0.01 and *** *p* < 0.001.

## 3. Results

### 3.1. Identification and Functional Analysis of ELIRF3

**Figure 1:**
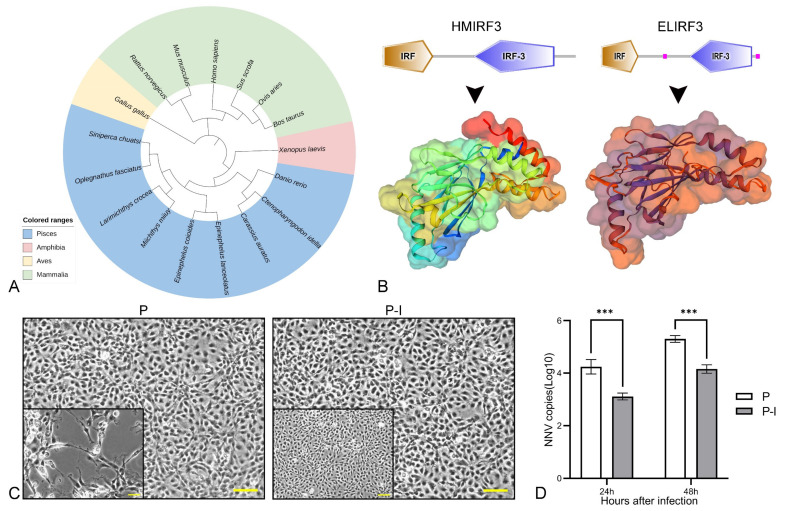
Bioinformatics analysis of ELIRF3 and validation of its antiviral function. Phylogenic analysis of ELIRF3 (**A**). Different colors represent different classes of animals, including *Aves* (light blue), *Mammalia* (green), *Amphibia* (pink), and *Pisces* (yellow). Prediction of protein domains and 3D models of *Homo sapiens* IRF3 (HMIRF3) and *Epinephelus lanceolatus* IRF3 (ELIRF3) (**B**). Cells (**C**) at 24 h after infection and qPCR results (**D**) of 24 h and 48 h after infection with NNV. Scale bar, 50 μm. P: pBudCE4.1 group; P-I: pBudCE4.1-IRF3 group. The data are presented as the means ± SD of three fish. Asterisks (*) on the bar represent the statistically significant differences, *** *p* < 0.001.

To analyze the evolutionary relationship between ELIRF3 and IRF3 in other species, a phylogenetic tree was constructed by the NJ method (Figure 1A). ELIRF3 is relatively closely related to most species of Pisces, but more distantly related to species of Aves and Mammalia. Schematic diagrams and 3D models of Homo sapiens IRF3 (HMIRF3) and ELIRF3 protein topologies were predicted using the SMART tool (Figure 1B). The two protein functional domains are similar, and the protein structures also have similar parts.

At 24 h and 48 h after infection, the qPCR results showed that the number of virus copies in the P group was significantly higher than that in the P-I group (Figure 1D), and the number of dead cells was also higher (Figure 1C). The above results show that ELIRF3 can significantly inhibit the replication of the virus at 24 h and 48 h of NNV infection, which also directly proves that IRF3 can be an immune adjuvant for NNV vaccines [25].

### 3.2. Construction and Identification of Recombinant Plasmids

**Figure 2:**
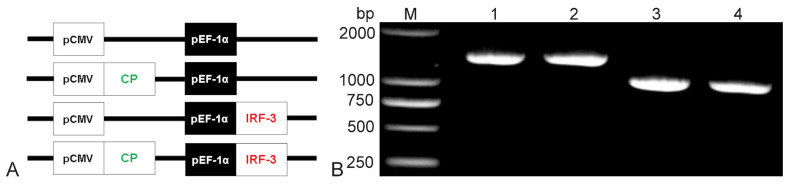
Construction of the bicistronic recombinant plasmid schematic structures of P, C-P, P-I, and C-P-I (**A**). The CP gene was inserted downstream of CMV promoter; the IRF3 gene was inserted following the EF-1α promoter. Results of agarose gel electrophoresis of recombinant plasmids PCR (**B**). Lane M: DL2000 DNA marker; Lane 1: P-I recombinant plasmid; Lane 2–3: C-P-I recombinant plasmid; Lane 4: C-P recombinant plasmid. P: pBudCE4.1 group; P-I: pBudCE4.1-IRF3 group; C-P: CP-pBudCE4.1 group; C-P-I: CP-pBudCE4.1-IRF3 group.

Bicistronic plasmids were constructed and illustrated with the help of a schematic diagram. The CP gene (1017 bp, antigenic gene from NNV) and the IRF3 gene (1374 bp, immune-adjuvant gene from grouper) were inserted into the pBudCE4.1 vector (Figure 2A). Then, the presence of the CP and IRF3 gene in the recombinant plasmids was confirmed by PCR (Figure 2B) which indicated the successful construction of the recombinant plasmids.

**Figure 3:**
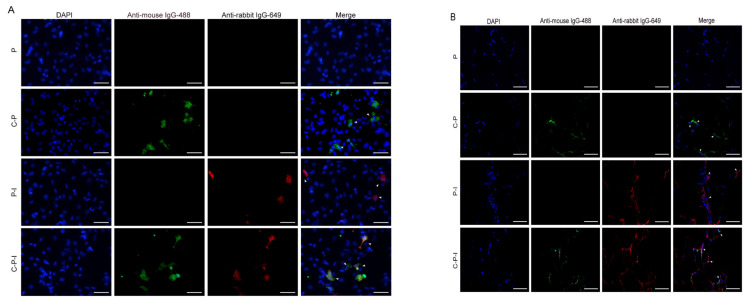
Expression of the bicistronic plasmids in vivo and in vitro. Protein expression in HINAE cells was analyzed by IFA 48 h after transfection (**A**). Muscles of fish five days after inoculation were cryosectioned (5 µm, (**B**)). Immunofluorescence labeling was performed by incubation with the mouse anti-NNV monoclonal antibody/rabbit anti-His polyclonal antibody and goat anti-mouse IgG-488/goat anti-rabbit IgG 649 to visualize the expression of CP and IRF3. The white arrow is the overlapped part of the fluorescent signal. Scale bar: 20 µm. P: pBudCE4.1 group; P-I: pBudCE4.1-IRF3 group; C-P: CP-pBudCE4.1 group; C-P-I: CP-pBudCE4.1-IRF3 group.

To investigate whether the CP and IRF3 gene were expressed in a eukaryotic system, the recombinant plasmids were transfected in HINAE cell line in vitro and injected into the grouper in vivo. In the results of the immunofluorescence analysis (IFA) of the transfected cells (Figure 3A) and cryosections of the muscle (Figure 3B), specific green fluorescence was observed in the C-P and C-P-I groups while red fluorescence was observed in the P-I and C-P-I groups. In contrast, no fluorescence was detected in the P group. The white arrows in the picture are the fluorescent overlap zone. The results revealed that the recombinant plasmids could successfully express CP and IRF3 proteins in vitro and in vivo, and can be used for subsequent immunization.

### 3.3. Expression of Immune-Related Genes in Spleen and Head Kidney after Immunization 

**Figure 4:**
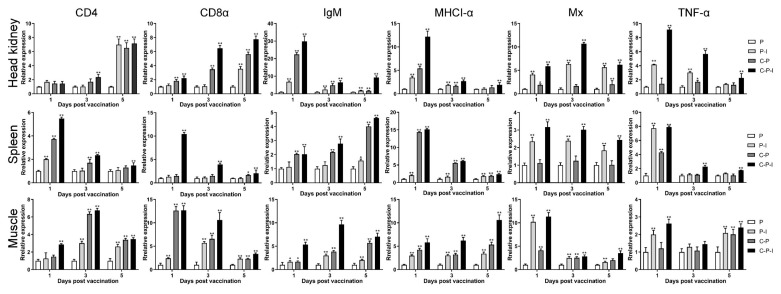
Expression changes of immune-related genes in head kidney, spleen, and muscle of injection site on the 1st/3rd/5th days after immunization. β-actin was used as an internal reference gene. The expression level of each gene in pBudCE4.1 group was set to one. The Y-axis represents the abundance of mRNA expression in fish relative to pBudCE4.1 in each group of fish. Results are shown as mean ± standard deviation of values randomly obtained from three fish. P: pBudCE4.1 group; P-I: pBudCE4.1-IRF3 group; C-P: CP-pBudCE4.1 group; C-P-I: CP-pBudCE4.1-IRF3 group. Asterisks (*) on the bar represent the statistically significant difference, * *p* < 0.05, ** *p* < 0.01.

The expression profiles of the immune-related genes at the spleen, head kidney, and muscle of the injection site were investigated by qPCR (Figure 4). The expression level of immune-related genes in pBudCE4.1 injected fish was set to one. Compared with this, all immune-related genes in the immune group were up-regulated to varying degrees, especially genes related to lymphocytes, such as CD4, CD8α, IgM, and MHCI-α, and some genes related to antiviral function, such as Mx and TNF-α.

In the spleen, except for IgM, which showed an upward trend, the expression of genes showed a downward trend in all groups during the experiment. Moreover, the expression of MHCI-α in the C-P and C-P-I group increased to approximately 14 and 15 times on the 1st day after immunization. The expression of TNF-α in the C-P and C-P-I group increased to approximately seven and eight times on the 1st day after immunization. In the head kidney: the expression of CD4 and CD8α showed an upward trend; IgM, MHCI-α, and TNF-α showed a downward trend; and Mx first rose and then fell. In addition, the expression of IgM in the C-P-I group increased to approximately 30 times on the 1st day after immunization. The expression of MHCI-α in the C-P-I group increased to approximately 12 times on the 1st day after immunization.

### 3.4. Local Immune Response of the Muscle at the Injection Site

In the muscles at the injection site (Figure 4), the expression of CD4 and IgM showed an upward trend and then a downward trend, the expression of MHCI-α showed an upward trend, the expression of CD8α and Mx showed a downward trend, and the expression of TNF-α remained unchanged. In addition, the expression of CD8α in the C-P and C-P-I group increased to approximately 12 and 13 times on the 1st day after immunization.

**Figure 5:**
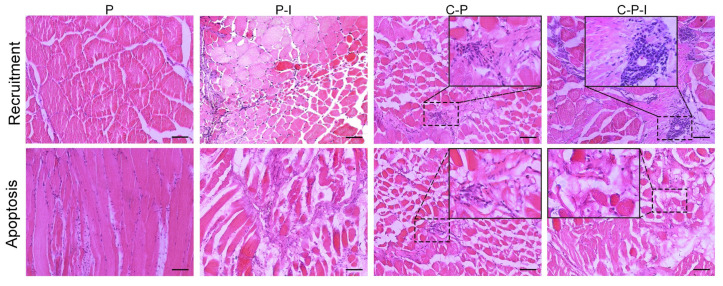
Histological changes in muscle after vaccination. Injection of plasmids leads to local recruitment of inflammatory cells and apoptosis of some muscle cells. Muscle sections (7 μm thick) obtained on 5th day following immunization of grouper with P, P-I, C-P, and C-P-I, respectively, were stained with hematoxylin and eosin. The picture in the box is a magnification of the corresponding area. P: pBudCE4.1 group; P-I: pBudCE4.1-IRF3 group; C-P: CP-pBudCE4.1 group; C-P-I: CP-pBudCE4.1-IRF3 group. Scale bar: 50 μm.

The recruitment of lymphocytes to the muscles at the injection site was investigated after plasmid injection in each group. After injection of C-P-I into the grouper, numerous lymphocytes were drawn to the site of injection on the 5th day, but few lymphocytes were observed in the control group injected with pBudCE4.1. The results for the 5th day are shown in Figure 5 (the data for 1st/3rd/7th day is not shown). In addition, some cells were apoptotic, and the apoptosis in the C-P-I group was significantly stronger than that in the P-I and C-P groups. The cytoplasm of the apoptotic cell became a pale pink, adhesion occurred, and the nucleus became smaller (the black box is the enlarged part).

### 3.5. Variations of the Percentage of sIgM+ Lymphocytes after Immunization

**Figure 6:**
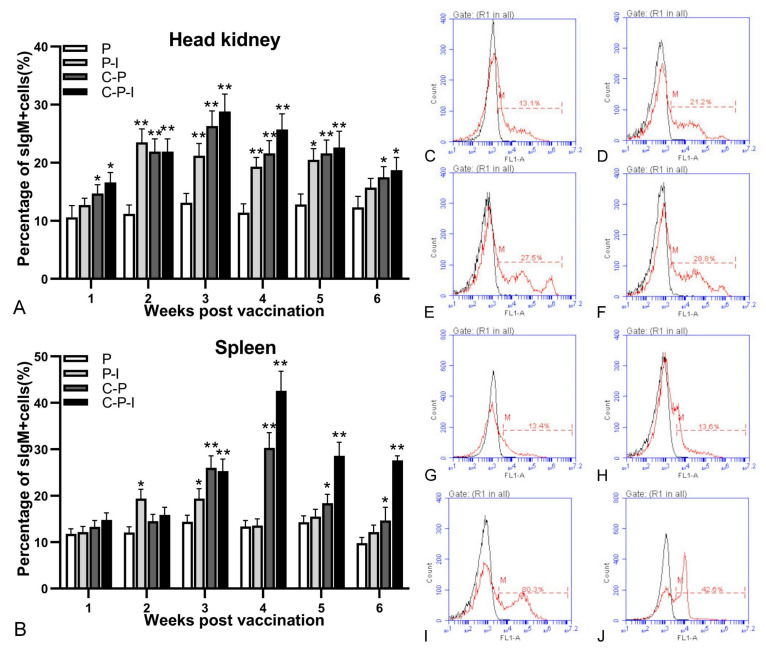
Variations of sIgM+ lymphocytes in head kidney and spleen. Changes in the percentage of sIgM+ lymphocytes in head kidney and spleen after immunization (**A**,**B**). Fluorescence histogram of gated lymphocytes (R1) showing the percentages of sIgM+ lymphocytes (scale of M) in the head kidney at the 3rd week post-immunization and spleen at the 4th week post-immunization of P, P-I, C-P, and C-P-I immunized fish (**C**–**J**). P: pBudCE4.1 group; P-I: pBudCE4.1-IRF3 group; C-P: CP-pBudCE4.1 group; C-P-I: CP-pBudCE4.1-IRF3 group. The data are presented as the means ± SD of three fish. Asterisks (*) on the bar represent the statistically significant difference, * *p* < 0.05, ** *p* < 0.01.

HKLs (Figure 6A) and SPLs (Figure 6B) of different groups grouper from the 1st to 6th weeks after inoculation, and changes of the percentage of sIgM+ lymphocytes were analyzed by FCM. The fluorescence histogram revealed the percentage of sIgM+ lymphocytes (red dotted line, M) in each group in the head kidney at the 3rd week and the spleen at the 4th week (Figure 6C–J). The percentage of sIgM+ lymphocytes in the control group remained almost unchanged. Compared with the control group, all three experimental groups could induce the proliferation of sIgM+ lymphocytes. Moreover, the percentage of sIgM+ lymphocytes of the P-I group showed a rapid increase in the 2nd week after immunization, and then decreased. In the head kidney, sIgM+ lymphocytes in all experimental groups were proliferated rapidly from 1st to 4th weeks. The percentage of sIgM+ lymphocytes of the C-P and C-P-I groups peaked (27.5 ± 2.6% and 28.8 ± 3.1%) in the 3rd week after immunization, and then declined, and finally there was little difference from the control group. In the spleen, lymphocytes in the C-P and C-P-I groups proliferated rapidly from 1st to 4th weeks and reached the highest percentage (30.3 ± 3.3% and 42.6 ± 4.2%) during the trial at 4th week.

### 3.6. Specific Antibody against NNV and Total Antibody in Serum after Immunization

**Figure 7:**
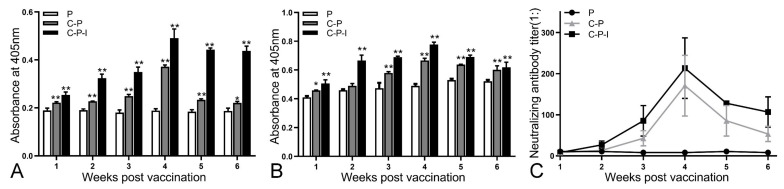
Changes of antibodies in serum from one to six weeks after immunization. Specific antibodies against NNV (**A**), total antibodies (**B**), and neutralizing antibody titers (**C**) from 1st to 6th weeks after immunization in vaccinated grouper were evaluated. P: pBudCE4.1 group; P-I: pBudCE4.1-IRF3 group; C-P: CP-pBudCE4.1 group; C-P-I: CP-pBudCE4.1-IRF3 group. The data are presented as the means ± SD of three fish. Asterisks (*) on the bar represent the statistically significant difference, * *p* < 0.05, ** *p* < 0.01.

The enhancement of anti-NNV specific antibody production by IRF3 was analyzed by ELISA using live NNV as the capture antigen. The level of antibodies in the control group remained stable during the experiment. All antibodies reached the highest level in the 4th week and then steadily declined. After inoculation with C-P and C-P-I plasmids, the antibody levels first increased, peaked in the 4th week, and then gradually decreased. At the 6th week after immunization, the levels of anti-NNV specific antibodies (Figure 7A) in the C-P and C-P-I groups were significantly higher than those in the P group (*p* < 0.05). In addition, the level of specific antibodies in the C-P-I group was significantly higher than that in the C-P and P groups in the 5th and 6th week. The changes of the level of total antibodies in the serum are similar to the level of specific antibodies (Figure 7B).

### 3.7. NNV Neutralizing Antibody Activity in Serum

The neutralizing ability of the serum antibodies isolated from each group of immunized fish was evaluated (Figure 7C). After immunization, the neutralizing antibody titer gradually increased with time and reached the highest level (1:256) in the 4th week after immunization. These were significantly higher than those obtained in the control group (** *p* < 0.01). In addition, the level of the C-P-I group was significantly higher than that of the C-P group.

### 3.8. RPS, Changes of sIgM+ Lymphocytes and Antibodies, Viral Load after Challenge

**Figure 8:**
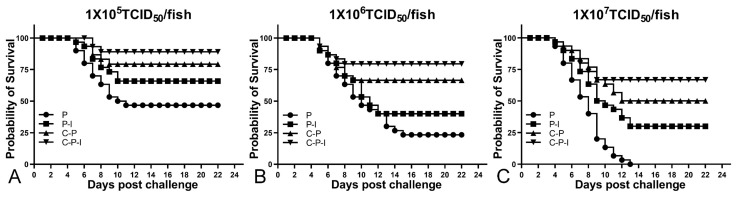
Survival curve of fish after challenge with three doses of virus (**A**): 1 × 10^5^ TCID_50_/fish group. (**B**): 1 × 10^6^ TCID_50_/fish group. (**C**): 1 × 10^7^ TCID_50_/fish group. P: pBudCE4.1 group; P-I: pBudCE4.1-IRF3 group; C-P: CP-pBudCE4.1 group; C-P-I: CP-pBudCE4.1-IRF3 group.

**Figure 9:**
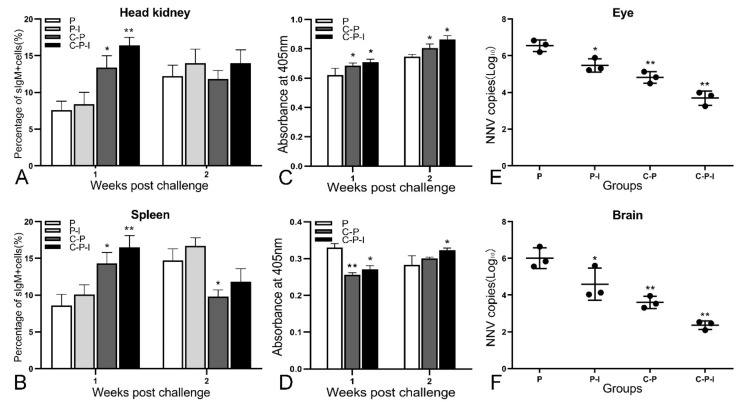
Immune protection provided by vaccination. In 1 × 10^6^ TCID_50_/fish group, the changes of percentage of sIgM+ lymphocytes in the head kidney (**A**) and spleen (**B**), the changes of total antibodies (**C**) and specific antibodies against NNV (**D**) in the serum for two weeks. Virus loads in eyes (**E**) and brains (**F**) of vaccinated grouper post-challenge with NNV. Results are expressed in log10 scale with mean viral load and standard deviation. P: pBudCE4.1 group; P-I: pBudCE4.1-IRF3 group; C-P: CP-pBudCE4.1 group; C-P-I: CP-pBudCE4.1-IRF3 group. Results are shown as mean ± standard deviation of values randomly obtained from three fish. Asterisks (*) on the bar represent the statistically significant difference, * *p* < 0.05, ** *p* < 0.01.

After the challenge, the fish mortality was monitored and the RPS was calculated; the RPS of the P-I, C-P, and C-P-I group were 37.5%, 62.5%, and 81.25% in 1 × 10^5^ TCID_50_/fish group; 30.43%, 56.52%, and 73.91% in 1 × 10^6^ TCID_50_/fish group; 30%, 50%, and 66.67% in 1 × 10^7^ TCID_50_/fish group (Figure 8). Compared with the C-P group, the RPS of the C-P-I group was improved, in 1 × 10^5^ TCID_50_/fish group, RPS increased by 18.75%, in 1 × 10^6^ TCID_50_/fish group, RPS increased by 17.39%, and in 1 × 10^7^ TCID_50_/fish group, RPS increased by 16.67%, indicating that IRF3 could be used as a molecular adjuvant to improve the immune protection effect of DNA vaccines. In the 1 × 10^6^ TCID_50_/fish group, the percentage of sIgM+ lymphocytes in the control group showed a trend of first declining and then rising, while the experimental group continued to decline (Figure 9A,B). As for the antibodies, the level of specific antibodies first increased and then decreased in the control group, while the level of total antibodies increased in the experimental group (Figure 9C,D). In addition, the copy number of NNV in brains and eyes of the experimental group was significantly lower than that of the control group (Figure 9E,F). The C-P-I group with the lowest virus copy number had a significant difference (*p* < 0.01) compared with the control group.

### 3.9. The Growth of the Fish after Immunization

**Figure 10:**
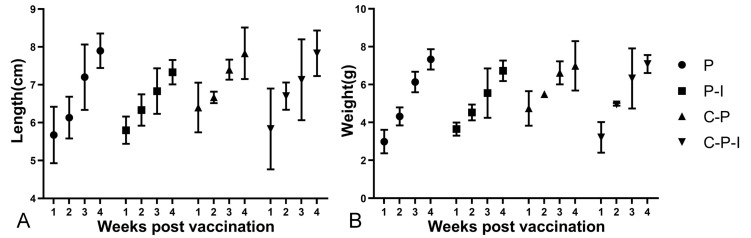
Changes in body length and weight of fish in each group from 1st to 4th weeks after immunization (**A**): Length (cm). (**B**): Weight (g). P: pBudCE4.1 group; P-I: pBudCE4.1-IRF3 group; C-P: CP-pBudCE4.1 group; C-P-I: CP-pBudCE4.1-IRF3 group. Results are shown as mean ± standard deviation of values randomly obtained from three fish.

To explore whether the vaccine had side effects on the growth of the fish, three fish were randomly selected every week to monitor the body length and weight to observe whether the growth rate had changed. As shown in Figure 10, the growth rates of the fish in each group are substantially similar, indicating that the vaccine has no side effects on the growth of the fish.

## 4. Discussion

The pBudCE4.1 plasmid is a dual eukaryotic expression vector that can simultaneously express two foreign genes under the control of the CMV and EF-1α promoters [26,27,28,29]. It is precisely because two segments of genes can be inserted that a bivalent vaccine or an immune adjuvant plus a monovalent vaccine can be made. In addition, some previous experiments have proved that the insertion of an antigen gene and a molecular adjuvant gene can improve the immune protection effect of the vaccine [21,30].

The innate immune system is the first line of defense against pathogens, and it is particularly important for juvenile fish whose immune system has not yet fully formed [31]. In vertebrates, interferon (IFN) exerts its antiviral function in the innate immune response mainly by inducing the expression of a variety of ISGs and regulating the expression of antiviral related proteins [32,33]. Interferon regulatory factors (IRFs) play an essential role in the innate antiviral response, mainly by acting as transcription factors that initiate interferon activation [34]. So far, 11 members of the IRF family have been identified in vertebrates, including 9 in mammals, 10 in birds, and 11 in fishes [35]. In addition, IRF3 has been proven to trigger the expression of IFN and ISGs in the early stages of viral infection [36]. Viral nucleic acid induces the phosphorylation of IRF3 and then causes the cytoplasmic to nuclear translocation of IRF3, which stimulates the transcription of IFN-I and other IFN-induced genes [13]. In this paper, we performed protein structure prediction and antiviral functional analysis of ELIRF3. The results showed that ELIRF3 is structurally similar to HMIRF3, and that ELIRF3 can inhibit NNV replication in the early stages of viral infection. The results of qPCR and CPE indicated that viral particles were inhibited at the genetic and cellular level, how IRF3 inhibits viral replication requires further study. Then, we inserted the CP gene of NNV and the IRF3 gene of pearl gentian grouper into the pBudCE4.1 vector under the control of the EF-1α and CMV promoters, respectively, to create bicistronic plasmids. After the transfection of the HINAE cell line and intramuscular injection, the IFA results showed that the constructed bicistronic plasmids can be successfully expressed in vivo and in vitro.

On the 5th day after immunization, the recruitment of lymphocytes was observed in the muscle of the injection site, and the expression of antigenic protein could also be detected, which could continuously stimulate the immune response in the fish, which has also been described in previous reports [21,22]. This provides some support for future exploration of the duration of vaccines. In the results of the H&E staining, the muscles at the injection site of the C-P, P-I, and C-P-I groups all showed apoptosis. It indicated that both CP and IRF3 could induce apoptosis, which is consistent with the previous reports [37,38,39]. This also indirectly indicates that the bicistronic plasmids have been successfully constructed and can be successfully expressed in vivo to exert their protein function. In addition, the expression of the antigen protein caused a certain degree of the recruitment of immune cells, which also led to the subsequent production of antibodies. The production of foreign protein induced the response of antigen uptake cells and antigen presenting cells, causing both cellular and humoral immunity, and then inducing the immune response of the fish. 

The spleen and head kidney are the main immune organs of fish. After immunization, the expression of T and B cell marker genes in the spleen and head kidney were up-regulated. This shows that the vaccine induced the humoral and cellular immunity of the fish. Moreover, the expression of MHC Iα was also up-regulated. Corresponding to the H&E staining results, the expression of the antigens induced the recruitment of immune cells and the response of antigen presenting cells. At the same time, the expression of several immune-related genes, such as TNF-α and Mx, were also up-regulated accordingly. In addition, sIgM+ lymphocytes proliferated from the 1st to 4th weeks after immunization, and reached a peak in the 3rd/4th week, which was consistent with the level of specific antibodies, total antibodies, and neutralizing antibodies in the serum.

In actual production, due to the large number of infected fish, the titer of the virus is very easy to change, which is a great challenge to vaccines. In our study, we used three titers of viruses, 10^5^, 10^6^, or 10^7^ TCID_50_/fish, which can better simulate most of the virus titers in practical applications, and can also provide a certain reference for actual production. In the three doses, the 10^6^ × TCID_50_/fish group was selected to investigate immune protection because of the similarity of all groups. It also can more comprehensively reflect the immune protection effect provided by the vaccine to the fish. The three titers of the virus caused different deaths in the three control groups, as did the experimental group. At the same time, in the 1 × 10^6^ TCID_50_/fish group, the changes in the specific antibodies, total antibodies, and percentage of sIgM+ lymphocytes, and the virus load of the brains and eyes after the challenge, were measured, in order to more accurately clarify the mechanism of the immune protection effect of the vaccine on the fish. After the challenge, the percentage of sIgM+ lymphocytes in the control group decreased first and then increased, while the experimental group continued to decline. The level of specific antibodies rose first and then fell in the control group, while in the experimental group it dropped first and then rose. The level of total antibodies increased in each group. The viral load was also measured in each group. However, the mechanism needs further experiments for clarification. The challenges of the pathogens with different titers, detection of cells, antibodies, and pathogen loads after the challenge also provides a point of reference for the improvement of the construction of a fish vaccine evaluation system.

There have been many reports on oral DNA vaccines [40,41,42]. Based on the immune protection effect of the vaccine in this paper, the oral bicistronic DNA vaccine needs further research. In addition, whether this vaccine can protect grouper against other viral infections like the previously reported VHSV DNA vaccine needs further experiments [12]. Moreover, there have been related reports indicating that the protrusion domain (P-domain) of the NNV capsid protein is related to virus entry into cells and antigenic properties. In future, the more immunogenic aspect of the capsid protein can be explored.

In this study, compared with the CP vaccine alone, the administration of bicistronic plasmids co-expressing IRF3 and CP protein increased the percentage of sIgM+ lymphocytes and the production of specific antibodies, and provided enhanced immune protection after the NNV challenge. The results revealed that IRF3 can be used as a molecular adjuvant for the CP DNA plasmid. In summary, the bicistronic DNA vaccine constructed in this article can provide new insight for the development of a DNA vaccine against NNV.

## Figures and Tables

**Table 1:** The sequences of primers used in this study.

Transcript	Sequence (5′–3′)	GenBank Acc. No
RNA2 (full)	F: CCAAGCTTATGGTACGCAAAGGTGAGAAG	MT157514.1
	R: CGGAATTCTTAGTTTTCCGAGTCAACCCT	
ELIRF3	F: GCGGCCGCATGTCTCATTCTAAACCAT	XM_ 033611218.1
	R: CGGGGTACCGTACATCTCCATCATCTCCTC	
CD4	F: TCCCACCTGAACAATCGTCC	HQ594532.1
	R: CACAGCTCACACCTCCACTT	
CD8α	F: GCTGGTGATTCTGCTGATTTG	GU124537.1
	R: GGACTTGGAGGATGACTTTAGG	
IgM	F: TACAGCCTCTGGATTAGACATTAG	HQ007252.2
	R: CTGCTGTCTGCTGTTGTCTGTGGAG	
Mx	F: TGAGGAGAAGGTGCGTCC	JX683389.1
	R: GCGCCTCCAACACGGAGCTC	
TNF-α	F: ACGCAATCAGGCCAAAGAG	AY667275.1
	R: AAGCCGCCCTGAGCAAAC	
MHC-Iα	F: CGACCTCACTCAGCATTGTCCT	FJ896112.3
	R: GTAGAAACCTGTAGCGTGGCG	
RNA2 (partial)	F: TGTGCCCCGCAAACAC	MT157514.1
	R: GACACGTTGACCACATCAGT	
β-actin	F: CCAGAGCAAGAGGGGTATC	KU200949.2
	R: GCTGTGGTGGTGAAGGAGT	

The underlined letters represent the restriction enzyme sites.

## Data Availability

The data presented in this study are available upon request from the corresponding author.
